# Ecological effects of sex differ with trophic positions in a simple food web

**DOI:** 10.1002/ece3.3740

**Published:** 2017-12-20

**Authors:** Kazutaka Kawatsu

**Affiliations:** ^1^ Department of Environmental Solution Technology Faculty of Science and Technology Ryukoku University Otsu Japan

**Keywords:** anisogamy, omnivory, persistence, predator–prey interaction, sexual difference, trophic flow

## Abstract

Sexual differences in parental investment, predation pressure, and foraging efforts are common in nature and affect the trophic flow in food webs. Specifically, the sexual differences in predator and prey behavior change in trophic inflow and outflow, respectively, while those in parental investment alter the reproductive allocation of acquired resources in the population. Consequently, these factors may play an important role in determining the system structure and persistence. However, few studies have examined how sexual differences in trophic flow affect food web dynamics. In this study, I show the ecological role of sex by explicitly incorporating sexual differences in trophic flow into a three‐species food web model. The results demonstrated that the ecological waste of males, that is, the amount of trophic inflow into males with less parental investment, plays an important role in system persistence and structure. In particular, the synergy between sexual differences in parental investment and trophic inflows and outflows is important in determining web persistence: Significant impacts of male‐biased trophic flows require the condition of anisogamy. In addition, the dynamic effects of the ecological waste of males differ with trophic level: The coexistence of a food web occurs more frequently with biased inflows into predator males, but occurs less frequently with biased inflows into consumer males. The model analysis indicates that investigating the pattern of sexual differences among trophic positions can enrich our understanding of food web persistence and structure in the real world.

## 1 INTRODUCTION

Coexisting species often have different niches and ecological traits, but they also share features, such as sex and the associated sexual differences: Many eukaryotic species reproduce sexually, and many aspects of their life differ between two sexes. First of all, anisogamy, in which one sex (mostly males) produces smaller gametes or invests less in an offspring than the other sex (mostly females), represents a fundamental sexual difference in two‐sex species (Clutton‐Brock, 1991). The anisogamy condition strengthens sexual selection for mates among males, which evolve diverse mating strategies, such as elaborate courtship dances in spiders, large horns in beetles, and decorative feathers in birds (Andersson, 1994). As sexual species are dominant in nature (Bell, 1982; Vrienhoek, 1998; White, 1973; Whitten, Sears, Baack, & Otto, 2008), it is interesting to ask what impacts the evolutionary corollary reproduces in the ecological perspective, such as species coexistence and community structure.

In ecological terms, sex and sexual differences alter trophic flow, which is a key driver of food web dynamics where organisms allocate resources acquired from prey to their survival and reproduction, in two ways. First, two‐sex population will allocate acquired resources to reproduction in very different way with a population of uniform sex, because parental investment into offspring in the broad sense is often less in males than in females among anisogamous species (Andersson, 1994; Clutton‐Brock, 1991; Trivers, 1972). Thus, sexual species often waste most of the resources flowing into males without investing in population recruitment (Lehtonen, Jennions, & Kokko, 2012). Second, sexual differences other than parental investment, such as increased body size, development of weapons, conspicuous appearance, and complex mating behaviors in one sex, may change the trophic flow in a predator–prey interaction directly. For example, these sexually selected traits may induce sex‐biased (usually male‐biased) mortality due to increased risks of predation and parasitism (e.g., Burk 1982; Boukal, Berec, & Krivan, 2008; Zuk & Kolluru, 1998). On the predator side, the development of large body sizes and exaggerated traits increases the requirement for resource use, which results in sex biases in foraging efforts (Rankin & Kokko, 2007). In fact, sex‐biased predation and parasitism are frequent in animals (Boukal et al., 2008) and plants (Cornelissen & Stilling, 2005; Marshal & Ganders, 2001), and there is plenty of information regarding sexually different foraging behavior in animals (Beck, Iverson, & Bowe, 2005; Morehouse, Nakazawa, Booher, Jeyasingh, & Hall, 2010; Mysterud, 2000; Ruckstuhl & Neuhaus, 2002; Tucker, Bowen, Iverson, Blanchard, & Stenson, 2009). In summary, while males less invest their resources into population growth, they may have some important ecological functions of trophic flows different to those of females. This suggests the importance of ecological effects of sexual differences in understanding food web dynamics and their outcomes.

Several theoretical investigations consider two‐sex dynamics explicitly. For example, Boukal et al. (2008) theoretically showed that sex‐selective predation (i.e., selective predation toward male or female prey) changes the stable coexistence of a predator–prey pair depending on the prey mating system. However, these studies dealt with two‐sex dynamics, either within a single‐species system (Castillo‐Chavez & Huang, 1995; Doebeli & Koella, 1994; Ruxton 1995; Lindström & Kokko, 1998) or only in the prey species when considering a system with two trophic levels (Boukal et al., 2008; Doebeli, 1997; Flatt, Marie, & Doebeli, 2001). I believe that these simplifications hinder our understanding of the ecological role of sex, especially of how the effects of sex differ with trophic positions, for the following reasons.

**Figure 1:**
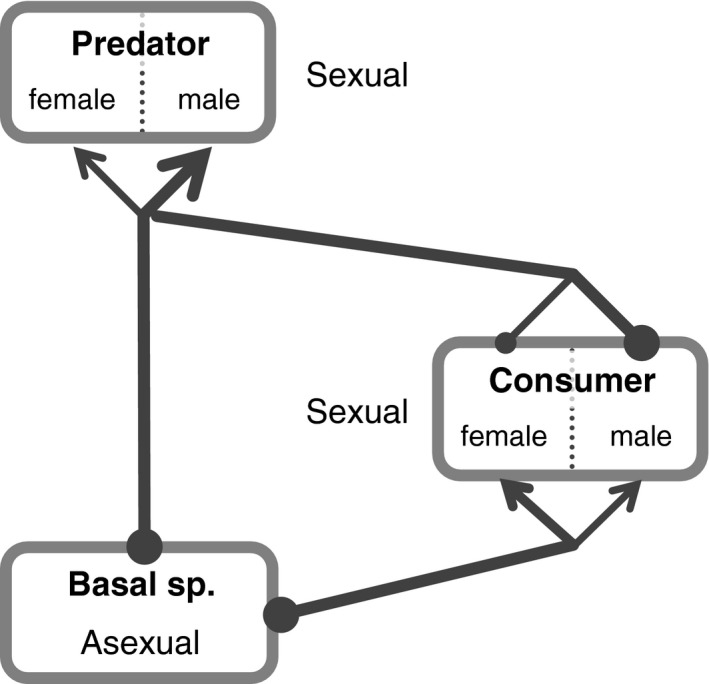
Scheme of the three‐species food web model with explicit sexual differences in trophic flow. Links connecting each species reflect the total trophic flow. Circles at the roots of links and arrows at the end of links indicate outflow and inflow of the population, respectively. The differences in the size of the circles and arrows within the same species indicate sexual differences in the trophic inflow and outflow

In sex‐explicit predator–prey dynamics, trophic flow can be divided into outflows from female prey and male prey, and inflows into female predators and male predators (Figure 1). Thus, sex biases in predation and foraging, working in synergy with sexual differences in parental investment, would change the dynamical properties of the system. For example, it is predicted that male‐biased predation in prey species with minimal parental investment of males might increase bottom‐up or donor control in food web dynamics: A decrease in prey males would have less impact on prey density than in females, but may sustain the trophic inflow into predators. On the other hand, male‐biased foraging might increase top–down or recipient control, because an increased inflow into male predators is reflected minimally in the population growth of the predator. In this study, to evaluate the ecological role of the sexes of predator and prey, I explicitly incorporate two‐sex dynamics into a simple food web model, which contains direct and indirect interspecific interactions. Then, I examine the effects of predator and prey sex on the persistence of the system separately, and the relative abundances of each species. The results of the model shed light on the ecological importance of sex according to trophic positions in nature.

## 2 MATERIALS & METHODS

### 2.1 Mathematical model

To investigate the ecological effects of predator and prey sex, let us start with a simple extension of a three‐species food web model consisting of a basal species, consumer, and top predator, to sexually explicit dynamics (Figure 1). In the model, because sexually reproducing species tend to be more frequently in higher trophic positions in nature (Bell, 1982; Clutton‐Brock, 1991; Vrienhoek, 1998; White, 1973; Whitten et al., 2008), the consumer and predator are heterotrophic sexual organisms, whereas the basal species is an autotrophic asexual organism. That is, the consumer and top predator used resources acquired from lower trophic organisms to recruit a new male and female population. I further extended the model to include the variation in mating systems (i.e., monogamy to polygyny) that real sexual organisms show (see below). In addition, I assume that the predator can eat the basal species as well as the consumer, to incorporate omnivory into the model. This is because this food web model is the simplest and best‐studied trophic module one in which both direct and indirect interspecific interaction co‐occur (e.g., Holt & Polis, 1997; Kondod, 2008; Polis, Myers, & Holt, 1989). Thus, this model would be useful to discuss the effect of sexual differences on the system persistence.

For the basal species, an autotrophic asexual organism, there are no demographic sexual differences. Assuming that species interactions occur simply in proportion to species densities, the dynamics of the basal species was modelled in the simplest form as follows:

(1) $$\frac{dB}{dt}=\left\{r-\left(s_{B}B+a_{\mathrm{BC}}\left(C_{f}+C_{m}\right)+a_{\mathrm{BP}}\left(P_{f}+P_{m}\right)\right)\right\}B,$$

where *B*,* C*_f_, *C*_m_, *P*_f_ and *P*_m_ stand for the density of the basal species, females and males of the consumer species and females and males of the top predator species, respectively. The parameters *r* and *s*_B_ are the intrinsic birth rate and self‐regulation intensity of the basal species, respectively. The parameters *a*_BC_ and *a*_BP_ indicate the rate of predation by the consumer and top predator, respectively.

Owing to heterotrophy and sexual reproduction, demographic sexual differences occur in the consumer and top predator. For illustrative purposes, I first defined species‐level trophic flows for the consumer and top predator. Specifically, although sex‐selective predation and sexual differences in foraging efforts may alter the strength of the predator–prey interaction, I assumed that such sexual differences do not alter the net trophic flow. This is because an increase in the net interaction strength, followed by changes in the female or male flow, simply affects the dynamics of predator–prey systems, and makes it difficult to evaluate the ecological effects of sexual differences correctly. Thus, the net trophic inflow into the consumer and top predator, and outflow from the consumer (*E*_C_, *E*_P_ and *D*_C_, respectively), depends on the species densities, as follows:

(2) $$\begin{array}{cc} E_{C} & =e_{\mathrm{CB}}a_{\mathrm{BC}}B\left(C_{f}+C_{m}\right)\\ E_{P} & =\left\{e_{\mathrm{PB}}a_{\mathrm{BP}}B+e_{\mathrm{PC}}a_{\mathrm{CP}}\left(C_{f}+C_{m}\right)\right\}\left(P_{f}+P_{m}\right)\\ D_{C} & =a_{\mathrm{CP}}\left(P_{f}+P_{m}\right)\left(C_{f}+C_{m}\right) \end{array}$$

where *e*_ij_ determines the trophic efficiency of species i at consuming prey j. The parameter *a*_CP_ is the predation rate of the consumer by the top predator. These net trophic flows are allocated to each sex depending on the population sex ratio weighted by the sexual difference parameter, as follows:

(3) $$\begin{array}{cc} E_{C,m} & =\frac{\gamma_{C}C_{m}}{C_{f}\;+\;\gamma_{C}C_{m}}E_{C}\\ E_{P,m} & =\frac{\gamma_{P}P_{m}}{P_{f}\;+\;\gamma_{P}P_{m}}E_{P}\\ D_{C,m} & =\frac{\delta_{C}C_{m}}{C_{f}\;+\;\delta_{C}C_{m}}D_{C} \end{array}$$

which assumes *E*_i _= *E*_i,f_ + *E*_i,m_ and *D*_i_ = *D*_i,f_ + *D*_i,m_ for inflow and outflow of species i, respectively. The parameter γ_i_ determines the relative male contribution to the net inflow of heterotrophic species i, and δ_C_ is the relative male contribution to the net outflow from the consumer.

During population recruitment by heterotrophic species, resources flowing into females and males are translated into reproducing daughters and sons in concert with the opposite sex. Although various functions have been used to describe the relative contributions of the two sexes to reproduction, I assumed that the birth rate of sexual species was proportional to the harmonic mean of the female and male densities, as well as the acquired resources (Caswell & Weeks, 1986; Miller & Inouye, 2011). The harmonic mean reproduction can be modified to handle the mating system of sexual species i using parameter *k*_i_ to describe the average male mating capacity (*k*_i _= 1 for strict monogamy and other values for polygyny, Caswell & Weeks, 1986; Lindström & Kokko, 1998). Specifically, the maximum reproductive contribution of each sex is proportional to the per capita inflow and the modified harmonic mean (e.g., *E*_C,f_/*C*_f_ × 2*C*_f_ *C*_m_/(*C*_f_ *k*_C_^−1^ + *C*_m_) for consumer female). In addition, to describe the sexual difference in parental investment, the male contribution to reproduction was weighted by parameter β_i_ for heterotrophic species i (0 ≤ β_i_ ≤ 1). With these considerations and the assumption of an equal sex ratio at birth, the two‐sex dynamics of the consumer and top predator becomes the following:

(4) $$\begin{array}{cc} \frac{dC_{f}}{dt} & =\frac{1}{2}\left(\frac{E_{c,f}}{C_{f}}+\beta_{C}\frac{E_{c,m}}{C_{m}}\right)\frac{2C_{f}C_{m}}{C_{f}k_{C}^{-1}\;+\;C_{m}}-D_{C,f}-s_{C}\left(C_{f}\;+\;C_{m}\right)C_{f}\\ & =\left\{\frac{e_{\mathrm{CB}}a_{\mathrm{BC}}B\left(1\;+\;\beta_{C}\gamma_{C}\right)C_{m}}{\left(C_{f}\;+\;\gamma_{C}C_{m}\right)\left(C_{f}k_{C}^{-1}\;+\;C_{m}\right)}-\left(\frac{a_{\mathrm{CP}}}{C_{f}\;+\;\delta_{C}C_{m}}\;+\;s_{C}\right)\right\}\left(C_{f}\;+\;C_{m}\right)C_{f}\\ \frac{dC_{m}}{dt} & =\frac{1}{2}\left(\frac{E_{c,f}}{C_{f}}\;+\;\beta_{C}\frac{E_{c,m}}{C_{m}}\right)\frac{2C_{f}C_{m}}{C_{f}k_{C}^{-1}\;+\;C_{m}}-D_{C,m}-s_{C}\left(C_{f}\;+\;C_{m}\right)C_{m}\\ & =\left\{\frac{e_{\mathrm{CB}}a_{\mathrm{BC}}B\left(1\;+\;\beta_{C}\gamma_{C}\right)C_{f}}{\left(C_{f}\;+\;\gamma_{C}C_{m}\right)\left(C_{f}k_{C}^{-1}\;+\;C_{m}\right)}-\left(\frac{\delta_{C}a_{\mathrm{CP}}}{C_{f}\;+\;\delta_{C}C_{m}}\;+\;s_{C}\right)\right\}\left(C_{f}\;+\;C_{m}\right)C_{m}\\ \frac{dP_{f}}{dt} & =\frac{1}{2}\left(\frac{E_{P,f}}{P_{f}}\;+\;\beta_{P}\frac{E_{P,m}}{P_{m}}\right)\frac{2P_{f}P_{m}}{P_{f}k_{P}^{-1}\;+\;P_{m}}-s_{P}\left(P_{f}\;+\;P_{m}\right)P_{f}\\ & =\left\{\frac{\left(e_{\mathrm{PB}}a_{\mathrm{BP}}B\;+\;e_{\mathrm{PC}}a_{\mathrm{CP}}\left(C_{f}\;+\;C_{m}\right)\right)\left(1\;+\;\beta_{P}\gamma_{P}\right)P_{m}}{\left(P_{f}\;+\;\gamma_{P}P_{m}\right)\left(P_{f}k_{P}^{-1}\;+\;P_{m}\right)}-s_{P}\right\}\left(P_{f}\;+\;P_{m}\right)P_{f}\\ \frac{dP_{m}}{dt} & =\frac{1}{2}\left(\frac{E_{P,f}}{P_{f}}\;+\;\beta_{P}\frac{E_{P,m}}{P_{m}}\right)\frac{2P_{f}P_{m}}{P_{f}k_{P}^{-1}\;+\;P_{m}}-s_{P}\left(P_{f}\;+\;P_{m}\right)P_{m}\\ & =\left\{\frac{\left(e_{\mathrm{PB}}a_{\mathrm{BP}}B\;+\;e_{\mathrm{PC}}a_{\mathrm{CP}}\left(C_{f}\;+\;C_{m}\right)\right)\left(1\;+\;\beta_{P}\gamma_{P}\right)P_{f}}{\left(P_{f}\;+\;\gamma_{P}P_{m}\right)\left(P_{f}k_{P}^{-1}\;+\;P_{m}\right)}-s_{P}\right\}\left(P_{f}\;+\;P_{m}\right)P_{m}, \end{array}$$

where *s*_C_ and *s*_P_ is the self‐regulation intensity of the consumer and top predator, respectively.

### 2.2 Model analysis

Unfortunately, I did not obtain an analytical solution for coexistence equilibria due to the complexity of the model. Instead, the system persistence and density of each species for the coexistence equilibria were analyzed by numerical simulations in the following manner. First, the intrinsic birth rate and self‐regulation intensity of the basal species were fixed as *r *=* *2 and *s*_B _= 1, respectively. The self‐regulation intensity of each trophic species was randomly determined from a uniform distribution *U*(0, 1). The rate of predation and the trophic efficiency of each heterotrophic species were also randomly assigned with *U*(0, 1). Sexual differences in foraging efforts (γ_C_ and γ_P_) and predation pressure (δ_C_) were varied from low (=0.5) to high (=5.0) male contribution. For sexual differences in parental investment, the male contribution to reproduction (β_C_ and β_P_) was assigned using 0.0, 0.5, or 1.0. The results were also compared between polygyny conditions (*k*_i _= 25) and strict monogamy conditions (*k*_i _= 1) in the heterotrophic species. The simulations were iterated 1,000 times for each parameter set. In each simulation run, the initial densities of the basal species, consumer, and top predator were randomly assigned from *U*(0, 1), and then the community dynamics were calculated for 100,000 time steps using the fourth‐order Runge–Kutta method with an integration step 0.01. The system persistence was evaluated as the proportion of runs where all of the species survived and the dynamics of each species reached its equilibrium (i.e., *X *>* *0.0 and ∂*X*/∂*t *≤* *1.0 × 10^−10^ for species *X*).

## 3 RESULTS

### 3.1 Effects of sexual differences in trophic flow on system persistence

**Figure 2:**
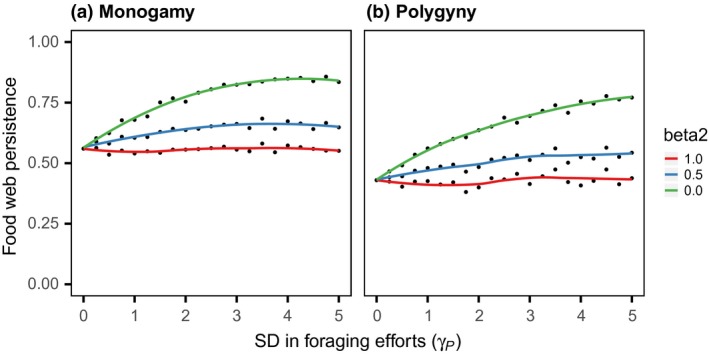
Relationship between the sexual difference in parental investment and the sexual difference of trophic flow in the top predator. The sexual difference in trophic inflow, γ_P_, was varied from 0.0 to 5.0, in increments of 0.25. Panels a and b are the results of monogamous and polygynous mating systems, respectively. Lines are the smoothing splines for the proportion of persistent food webs, performed with the ‘smooth.spline’ function of R software (ver. 3.3.2) (red: β_P_ = 1.0; blue: β_P* *_= 0.5; green: β_P* *_= 0.0). The other parameters are explained in the text

**Figure 3:**
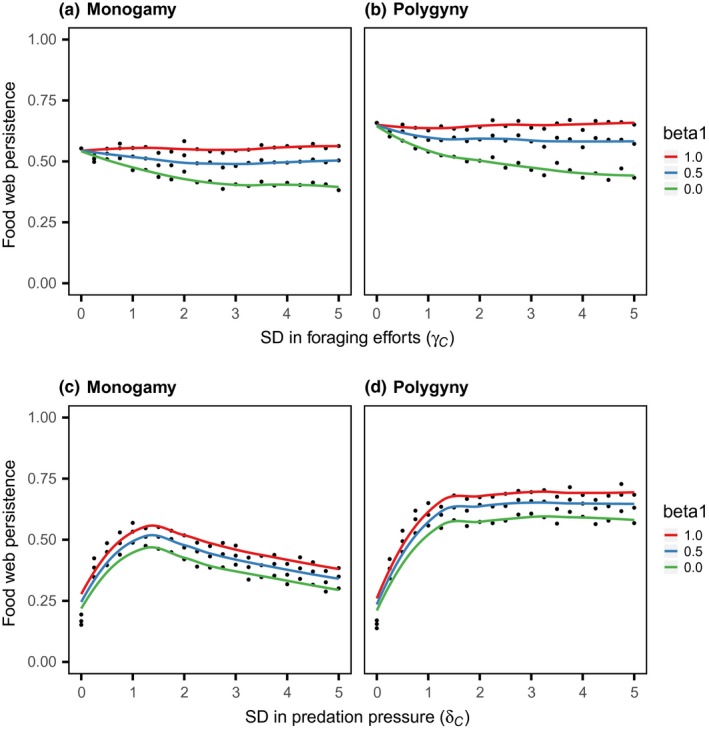
Relationship between the sexual difference in parental investment and the sexual difference in trophic flow of the consumer. The top (a and b) and bottom (c and d) panels are the results for the sexual difference in trophic inflow, γ_C_, and trophic outflow, δ_C_, respectively (both were varied from 0.0 to 5.0, in increments of 0.25). The left (a and c) and right (b and d) panels are the results for monogamous and polygynous mating systems, respectively. The lines are the smoothing splines for the proportion of persistent food webs with the ‘smooth.spline’ function of R ver 3.3.2 (red: β_C_ = 1.0; blue: β_C* *_= 0.5; green: β_C* *_= 0.0). The other parameters are explained in the text

The numerical simulations demonstrated that sexual differences in the top predator affect food web persistence (Figure 2). Specifically, with less male contribution to population growth (i.e., β_P _< 1.0), male‐biased trophic inflow improved the food web persistence. This demographic effect of a sexual difference in foraging was maximized under the condition of minimum paternal investment (i.e., β_P _= 0.0), but was lost under the isogamous condition (β_P _= 1.0). Regarding the difference in the predator's mating system, male/sperm limitation or strict monogamy improved the system persistence, but did not change the effect of the sexual difference in foraging qualitatively. Sexual differences in the consumer affected the food web persistence in a manner different to the effect of the predator (Figure 3). Specifically, male‐biased trophic inflow decreased the food web persistence when there was a reduced male contribution to population growth (β_C _< 1.0). The negative effect of the sexual difference in the consumer's foraging efforts was also maximized under the condition of minimum paternal investment, but was lost under the isogamy condition. Regarding differences in the consumer's mating system, strict monogamy decreased the system persistence but did not change the effect of sexual difference in foraging qualitatively (Figure 3a,b). For the sexual difference in predation attack, the food web persistence improved initially with an increased male contribution to trophic outflow, but ultimately decreased in strict monogamy (Figure 3c) or became saturated under the polygyny condition (Figure 3d). There was no interaction effect between the sexual difference in the consumer's predation attack and that in the consumer's parental investment.

### 3.2 Synergy in sexual differences

**Figure 4:**
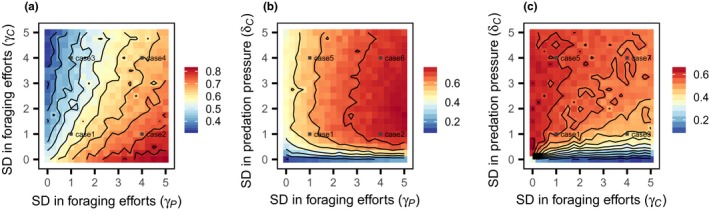
The interaction effect between trophic flow on food web persistence. The results of the interaction between (a) the inflow sexual difference in the predator (γ_P_) and consumer (γ_C_), (b) the inflow sexual difference in the predator (γ_P_) and the outflow sexual difference in the consumer (δ_C_), and (c) the inflow sexual difference (γ_C_) and outflow sexual difference (δ_C_) in the consumer. The different colors indicate the proportion of persistent food webs in the numerical simulation (contour lines connect parameter regions showing the same persistence). All of the sexual differences were varied from 0.0 to 5.0 by 0.25. The other parameters are explained in the text

The above analysis demonstrated that the ecological effects of sexual differences in trophic flow depend on the condition of anisogamy. Then, further simulations were performed to investigate whether each sexual difference in trophic flow acts, in synergy with the sexual difference in the other flow, on the system persistence. The simulations were performed under the condition of complete anisogamy (β_C _= β_P_ = 0.0), and the results are summarized in Figure 4. For the interaction between sexual differences in the foraging efforts of predator (γ_P_) and consumer (γ_C_), there was an antagonistic effect on system persistence (Figure 4a). That is, the system persistence improved as the predator's trophic inflow became male‐biased, but this effect was depressed as the prey's inflow became biased toward males. For the interaction between sexual differences in predator foraging efforts and prey predation pressure (δ_C_), there was an additive effect on system persistence (Figure 4b). The food web persistence initially increased as the prey's outflow was biased toward males, but extreme male biases suppressed this increase. Although a male bias in the predator's inflow improved the food web persistence, it did not alter the effect of sexual differences in the prey's inflow qualitatively. Concerning the relationship between sexual differences in prey foraging efforts and predation pressure, the interaction appeared to be more complicated. Specifically, a male bias in sexual differences in inflow had a negative effect on food web persistence, but this effect weakened with a bias in outflow toward males (Figure 4c).

### 3.3 Effects of sexual differences on food web structure

**Figure 5:**
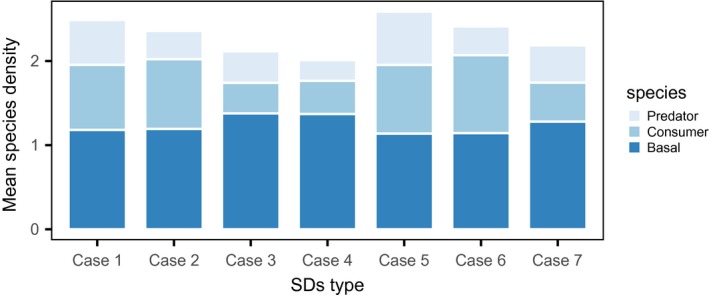
The effects of sexual differences on the species densities on persistent equilibria. The stacked bars indicate the mean equilibrium density of each species. The sexual difference parameters used in each case (1–7) are noted in Figure 4. The other parameters are explained in the text

The simulation analysis demonstrated that trophic sexual differences also affected the density of species at their equilibria (Figure 5). Specifically, compared with the case with no trophic sexual differences (case 1), male‐biased inflows at each trophic level similarly reduced their own density, but had differential impacts on the other species: Those in the top predator had no effect on the basal species, but increased the consumer density (case 2), while those in the consumer increased the basal species and decreased the top predator density (case 3). The male‐biased outflows in the consumer decreased the density of the basal species, but increased both heterotrophic species (case 5). The analysis also showed that each trophic sexual difference acted additively in determining the species densities. With male‐biased inflows in the consumer and top predator (case 4), the top predator had the lowest density, but the consumer density was slightly higher than that of case 3. With male‐biased inflow in the top predator and male‐biased outflow in the consumer (case 6), the consumer had the greatest density, while the others had densities similar to those in case 2. With male‐biased inflows and outflows in the consumer (case 7), the density of each species was the average of case 3 and 5.

## 4 DISCUSSION

Sex and associated sexual differences are prevalent in natural communities and are a determinant of the trophic flow of food webs; however, most ecological models have assumed a population of uniform sex (Caswell, 2001), and two‐sex dynamics have been investigated only in single‐species systems (Doebeli & Koella, 1994; Castillo‐Chavez & Huang, 1995; Lindström & Kokko, 1998; Ruxton 2008) or on the prey side of two trophic‐level systems (Boukal et al., 2008; Doebeli, 1995; Flatt et al., 2001). Here, I demonstrated the ecological role of sex in food web dynamics by incorporating sexual differences in trophic flow explicitly into a three‐species model. In particular, the model analysis suggested that the ecological waste of males, that is, the amount of trophic inflow into males with less parental investment, plays an important role in system persistence and structure.

Lessons from evolutionary theory tell us that trophic inflow into males is reflected minimally in population recruitment in anisogamous organisms, because males contribute less to parental investment than do females. Superficially, this only dampens the population growth rate, but the analysis revealed that the ecological waste of males alters food web persistence in concert with sexual differences in trophic flow. For example, male‐biased trophic inflow affects persistence only with a reduced male contribution to population growth (Figures 2 and 3a,b). In addition, this dynamical effect differs by trophic positions: The food web dynamics becomes robust with biased inflows into prey males, but vulnerable with those into consumer males. This is consistent with the previous analysis of the three‐species omnivory model without two‐sex dynamics: The system becomes more robust when the prey species can outcompete the predator for the resource use (Holt & Polis, 1997). The analysis also demonstrated that sexual differences in trophic flow act synergistically to affect system persistence (Figure 4). These results were qualitatively consistent, regardless of the mating system of the sexual species (Figures 2 and 3), and indicate that the distribution of sexual differences in trophic flow, if any, should play a critical role in food web persistence in nature.

The model analysis found that sexual differences in trophic flow also affected food web structure (Figure 5), that is, male‐biased inflows decrease their density and increase the density of species belonging to a lower trophic position, while male‐biased outflow in the consumer increases the density of that consumer and top predator. These results should also stem from the ecological waste of males. That is, trophic inflow into males spends resources that should have been available for population growth because males less invest into population growth. Therefore, any biases in trophic inflow toward males should increase this cost; increasing predation pressure on males in contrast releases this cost because resources should remain free from being consumed by wasteful males. These changes in species density due to the waste of males may make redundant resources available for others at the same trophic position, and might be related to mechanisms involved in the maintenance of biodiversity. Many theoretical and empirical studies have explored the mechanisms determining food web structure (e.g., Hairston, Smith, & Slobodkin, 1960; Murdoch, 1966; Paine, 1980; Schmitz, Hamback, & Beckerman, 2000). To my knowledge, however, little work in the view of sexual difference has been examined , and further theoretical studies are needed to investigate the effect of sex biases in trophic flows on structure and maintenance mechanisms of food webs.

Present paper studied the ecological effect of sexual differences in a simple, three‐species model with omnivory (like an intraguild predation module). This approach analyzing the dynamics of simple trophic modules, often consisting of three or four species (Hairston et al., 1960; Hastings & Powell, 1991; McCann, Hastings, & Huxel, 1998), is useful to understand the ecological function of interspecific interactions in food webs (Holt & Polis, 1997). In real nature, however, so many coexisting species interact with each other and there would be no isolated modules. In fact, an empirical study showed that a trophic module embedded in a larger real food web has an effect on the food web persistence different to the prediction from theoretical, isolated modules (Kondod, 2008). In an analogous way, complex interspecific interactions and the interaction of the different modules may change the ecological effect of sexual differences. Thus, it is interesting and open question to investigate how sexual differences affect the structure and persistence of large ecological communities.

In summary, the results of the model analysis demonstrate that the distribution of sexual differences in trophic flow among different trophic positions plays a critical role in food web persistence and structure. The literature indicates that sexually reproducing anisogamous organisms tend to occupy higher trophic positions, and asexual reproduction and isogamy occur more frequently in autotrophic organisms, such as plants, than in heterotrophic animals (Bell, 1982; Clutton‐Brock, 1991; Vrienhoek, 1998; White, 1973; Whitten et al., 2008). For more specific example, a field survey reported that sexually reproducing species appeared more frequently in higher trophic positions of oribatid mite communities (Fischer, Meyer, & Maraun, 2014). A similar trend should be observed in polar zooplankton communities: Asexual reproduction occurs more frequently in herbivorous zooplankton species than in omnivorous and carnivorous ones (Hagen, 1999). In addition, given that sexual selection shapes different adaptations in many physiological and behavioral traits between two sexes, sexual differences in predation, parasitism, and foraging effort are common in animals (e.g., Beck et al., 2005; Boukal et al., 2008; Cornelissen & Stilling, 2005; Marshal & Ganders, 2001; Morehouse et al., 2010; Mysterud, 2000; Paiva, Pereira, Ceia, & Ramos, 2017; Roy, Seehausen, & Nosil, 2013; Ruckstuhl & Neuhaus, 2002; Tucker et al., 2009). However, we lack information on how trophic positions and sex biases in trophic flows are correlated in the real world. Thus, further studies of the pattern of sexual differences among trophic positions would enrich our understanding of food web persistence and structure in nature.

## CONFLICT OF INTEREST

None declared.

## References

[ece33740-bib-0001] Andersson, M. (1994). Sexual selection. Princeton, NJ: Princeton University Press.

[ece33740-bib-0002] Beck, A. C. , Iverson, S. J. , & Bowe, W. D. (2005). Blubber fatty acids of gray seals reveal sex differences in the diet of a size‐dimorphic marine carnivore. Canadian Journal of Zoology, 83, 377–388. https://doi.org/10.1139/z05-021

[ece33740-bib-0003] Bell, G. (1982). The masterpiece of nature: The evolution of genetics and sexuality. London, UK: Croon Helm.

[ece33740-bib-0004] Boukal, D. S. , Berec, L. , & Krivan, V. (2008). Does sex‐selective predation stabilize or destabilize predator‐prey dynamics? PLoS ONE, 3, e2678. 18628951 10.1371/journal.pone.0002687 PMC2444021

[ece33740-bib-0500] Burk, T. , (1982). Evolutionary significance of predation on sexually signalling males. Florida Entomologist, 65, 90–104.

[ece33740-bib-0005] Castillo‐Chavez, C. , & Huang, W. (1995). The logistic equation revisited: The two‐sex case. Mathematical Biosciences, 128, 299–316. https://doi.org/10.1016/0025-5564(94)00077-D 7606140 10.1016/0025-5564(94)00077-d

[ece33740-bib-0006] Caswell, H. (2001). Matrix population models. Sunderland, MA: Sinauer.

[ece33740-bib-0007] Caswell, H. , & Weeks, D. E. (1986). Two‐sex models: Chaos, extinction, and other dynamic consequences of sex. The American Naturalist, 128, 707–735. https://doi.org/10.1086/284598

[ece33740-bib-0008] Clutton‐Brock, T. H. (1991). The evolution of parental care. Princeton, NJ: Princeton University Press.

[ece33740-bib-0009] Cornelissen, T. , & Stilling, P. (2005). Sex‐biased herbivory: A meta‐analysis of the effects of gender on plant‐herbivore interactions. Oikos, 111, 488–500. https://doi.org/10.1111/j.1600-0706.2005.14075.x

[ece33740-bib-0010] Doebeli, M. (1997). Genetic variation and persistence of predator‐prey interaction in the Nicholson‐Bailey model. Journal of Theoretical Biology, 188, 109–120. https://doi.org/10.1006/jtbi.1997.0454

[ece33740-bib-0011] Doebeli, M. , & Koella, J. C. (1994). Sex and population dynamics. Proceedings of the Royal Society B, 257, 17–23. https://doi.org/10.1098/rspb.1994.0088

[ece33740-bib-0012] Fischer, B. M. , Meyer, E. , & Maraun, M. (2014). Positive correlation of trophic level and proportion of sexual taxa of origabit mites (Acari: Oribatida) in alpine soil systems. Experimental and Applied Acarology, 63, 465–479. https://doi.org/10.1007/s10493-014-9801-3 24687174 10.1007/s10493-014-9801-3

[ece33740-bib-0013] Flatt, T. , Marie, N. , & Doebeli, M. (2001). A bit of sex stabilizes host‐parasite dynamics. Journal of Theoretical Biology, 212, 345–354. https://doi.org/10.1006/jtbi.2001.2380 11829355 10.1006/jtbi.2001.2380

[ece33740-bib-0014] Hagen, W. (1999). Reproductive strategies and energetic adaptations of polar zooplankton. Invertebrate Reproduction & Develoopment, 36, 25–34. https://doi.org/10.1080/07924259.1999.9652674

[ece33740-bib-0015] Hairston, N. G. , Smith, F. E. , & Slobodkin, L. B. (1960). Community structure, population control, and competition. The American Naturalist, 44, 421–425. https://doi.org/10.1086/282146

[ece33740-bib-0016] Hastings, A. , & Powell, T. (1991). Chaos in a three‐species food chain. Ecology, 72, 896–903. https://doi.org/10.2307/1940591

[ece33740-bib-0017] Holt, R. D. , & Polis, G. A. (1997). A theoretical framework for intraguild predation. The American Naturalist, 149, 745–764. https://doi.org/10.1086/286018

[ece33740-bib-0018] Kondod, M. (2008). Building trophic modules into a persistent food web. Proceedings of the National Academy of Science USA, 105, 16631–16635. https://doi.org/10.1073/pnas.0805870105 10.1073/pnas.0805870105 PMC2570427 18936484

[ece33740-bib-0019] Lehtonen, J. , Jennions, M. D. , & Kokko, H. (2012). The many cost of sex. Trends in Ecology and Evolution, 27, 172–178. https://doi.org/10.1016/j.tree.2011.09.016 22019414 10.1016/j.tree.2011.09.016

[ece33740-bib-0020] Lindström, J. , & Kokko, H. (1998). Sexual reproduction and population dynamics: The role of polygyny and demographic sex differences. Proceedings of the Royal Society B, 256, 483–488. https://doi.org/10.1098/rspb.1998.0320 10.1098/rspb.1998.0320 PMC1688919 9606132

[ece33740-bib-0021] Marshal, M. , & Ganders, F. R. (2001). Sex‐biased seed predation and the maintenance of females in gynodioecious plant. American Journal of Botany, 88, 1437–1443. https://doi.org/10.2307/3558451 21669676

[ece33740-bib-0022] McCann, K. , Hastings, A. , & Huxel, G. R. (1998). Weak trophic interactions and the balance of nature. Nature, 395, 794–798. https://doi.org/10.1038/27427

[ece33740-bib-0023] Miller, T. E. X. , & Inouye, B. D. (2011). Confronting two‐sex demographic models with data. Ecology, 92, 2141–2415. https://doi.org/10.1890/11-0028.1 22164838 10.1890/11-0028.1

[ece33740-bib-0024] Morehouse, N. I. , Nakazawa, T. , Booher, C. M. , Jeyasingh, P. D. , & Hall, M. D. (2010). Sex in a material world: Why the study of sexual reproduction and sex‐specific traits should become more nutritionally‐explicit. Oikos, 119, 766–778. https://doi.org/10.1111/j.1600-0706.2009.18569.x

[ece33740-bib-0025] Murdoch, W. W. (1966). Community structure, population control and competition‐a critique. The American Naturalist, 100, 219–226. https://doi.org/10.1086/282415

[ece33740-bib-0026] Mysterud, A. (2000). The relationship between ecological segregation and sexual body size dimorphism in large herbivores. Oecologia, 124, 40–54. https://doi.org/10.1007/s004420050023 28308411 10.1007/s004420050023

[ece33740-bib-0027] Paine, R. T. (1980). Food webs: Linkage, interaction strength, and community infrastructure. Journal of Animal Ecology, 49, 667–685.

[ece33740-bib-0028] Paiva, V. H. , Pereira, J. , Ceia, F. R. , & Ramos, J. A. (2017). Environmentally driven sexual segregation in a marine top predator. Scientific Reports, 7, 2590 https://doi.org/10.1038/s41598-017-02854-2 28572630 10.1038/s41598-017-02854-2 PMC5453963

[ece33740-bib-0029] Polis, G. A. , Myers, C. A. , & Holt, R. D. (1989). The ecology and evolution of intraguild predation: Potential ecological networks. Ecology Letters, 8, 1317–1325.

[ece33740-bib-0030] Rankin, D. J. , & Kokko, H. (2007). Do males matter? The role of males in population dynamics. Oikos, 116, 335–348. https://doi.org/10.1111/j.0030-1299.2007.15451.x

[ece33740-bib-0031] Roy, D. , Seehausen, O. , & Nosil, P. (2013). Sexual dimorphism dominates divergent host plant use in stick insect trophic morphology. BMC Evolutionary Biology, 13, 135 https://doi.org/10.1186/1471-2148-13-135 23819550 10.1186/1471-2148-13-135 PMC3707739

[ece33740-bib-0032] Ruckstuhl, K. E. , & Neuhaus, P. (2002). Sexual segregation in ungulates: A comparative test of three hypotheses. Biological Review, 77, 77–96. https://doi.org/10.1017/S1464793101005814 10.1017/s1464793101005814 11911375

[ece33740-bib-0600] Ruxton, G. D. , (1995). Population models with sexual reproduction show a reduced propensity to exhibit chaos. Journal of Theoretical Biology, 175, 595–601.

[ece33740-bib-0033] Schmitz, O. J. , Hamback, P. A. , & Beckerman, A. P. (2000). Trophic cascades in terrestrial systems: A review of the effects of carnivore removals on plants. The American Naturalist, 155, 141–153. https://doi.org/10.1086/303311 10.1086/303311 10686157

[ece33740-bib-0034] Trivers, R. L. (1972). Parental investment and sexual selection In Campbell E. (Ed.), Sexual selection and the descent of man (pp. 136–179). Chicago, IL: Aldine Publishing.

[ece33740-bib-0035] Tucker, S. , Bowen, W. D. , Iverson, S. J. , Blanchard, W. , & Stenson, G. B. (2009). Sources of variation in diets of harp and hooded seals estimated from quantitative fatty acid signature analysis (QFASA). Marine Ecology Progress Series, 384, 287–302. https://doi.org/10.3354/meps08000

[ece33740-bib-0036] Vrienhoek, R. C. (1998). Animal clones and diversity. BioScience, 48, 617–628. https://doi.org/10.2307/1313421

[ece33740-bib-0037] White, M. J. D. (1973). Animal ecology and evolution. Cambridge, UK: Cambridge University Press.

[ece33740-bib-0038] Whitten, J. , Sears, C. J. , Baack, E. J. , & Otto, S. P. (2008). The dynamic nature of apomixes in the angiosperms. International Journal of Plant Science, 169, 169–182. https://doi.org/10.1086/523369

[ece33740-bib-0039] Zuk, M. , & Kolluru, G. R. (1998). Exploitation of sexual signals by predators and parasitoids. The Quarterly Review of Biology, 73, 415–438. https://doi.org/10.1086/420412

